# Changes in the serotype distribution of *Streptococcus pneumoniae* causing otitis media after PCV13 introduction in Spain

**DOI:** 10.1371/journal.pone.0209048

**Published:** 2018-12-18

**Authors:** María Morales, Guillermo Ludwig, Maria Ercibengoa, Cristina Esteva, Viviana Sanchez-Encinales, Marta Alonso, Carmen Muñoz-Almagro, José Maria Marimón

**Affiliations:** 1 Donostia University Hospital–Biodonostia Health Research Institute, Donostia-San Sebastian, Spain; 2 CIBER Enfermedades Respiratorias (CIBERES), Instituto de Salud Carlos III, Madrid, Spain; 3 Molecular Microbiology Department, Institut de Recerca Sant Joan de Déu, Sant Joan de Déu University Hospital, Barcelona, Spain; 4 Preventive Medicine and Health Public Department, University of the Basque Country (UPV/EHU), Donostia-San Sebastian, Spain; 5 CIBER de Epidemiología y Salud Pública (CIBERESP), Instituto de Salud Carlos III, Madrid, Spain; 6 School of Medicine, Universitat Internacional de Catalunya, Barcelona, Spain; Universidade de Lisboa Faculdade de Medicina, PORTUGAL

## Abstract

One of the beneficial effects of pneumococcal conjugate vaccines (PCVs) has been a decrease in the incidence of non-invasive infections, such as otitis media (OM) caused by vaccine serotypes. In this study, we analyzed the epidemiology of pneumococcal OM before and after PCV13 introduction in 2010. Between 2008 and 2016, the middle ear exudates from 2653 children under 14 years of age with OM were studied in two Spanish provinces (Gipuzkoa and Barcelona), and *S*. *pneumoniae* was isolated in 235 (8.9%) of cases. The 204 available isolates were serotyped and distributed in three 3-year periods: one before and two after PCV13 introduction (early and late post-PCV13). A significant decrease in the rate of OM caused by *S*. *pneumoniae* was observed mainly due to a decrease in infections caused by all PCV13 serotypes, although exceptions were observed including the persistence of serotype 3 in Gipuzkoa and a weak re-emergence of serotype 19F in both regions. The rate and diversity of non-PCV13 serotypes increased in both regions and an emerging clone causing OM was detected in each region: serotype 23B ST2372 in Gipuzkoa and serotype 11A ST838/ST6521 in Barcelona. The introduction of PCV13 has been followed by a change in the epidemiology of pneumococcal OM, with a decrease in the rate of vaccine serotypes accompanied by an increase in the diversity of non-vaccine serotype and the clonal spreading of different single clones in each region.

## Introduction

Acute otitis media (OM) is an important health and economic problem since it is one of the main reasons for health care center visits, antibiotic prescriptions and pediatric surgical procedures [1–3]. Between 50% and 85% of children will have had one or more episodes of OM during their first 3 years of life and 40% six or more during their first 7 years of life [2]. *Haemophilus influenzae* and *Streptococcus pneumoniae* are the main bacteria responsible for OM. *Moraxella catarrhalis*, *Streptococcus pyogenes* and *Staphylococcus aureus* are also commonly found to be causing OM [4–6]. In particular, it is estimated that pneumococcus produces 30–50% (300 million per year) of all cases of acute OM worldwide [7]. *S*. *pneumoniae* can colonize the human nasopharynx from the first weeks of life reaching colonization rates as high as 89.5% in under-3-year-olds [8]. Once carriage status is established, pneumococcal ascension through the Eustachian tubes can lead to an initial episode of OM. Recurrent OM episodes are often associated with polymicrobial infections (*S*. *pneumoniae*, *H*. *influenzae* and *M*. *catarrhalis*) and the formation of biofilm which increases the persistence of the infection [9]. Early pneumococcal OM episodes are caused by serotypes commonly associated with invasive disease, while recurrences are more often associated with less invasive serotypes which are more prone to produce mixed infections and biofilm formation [10–12].

The polysaccharide capsule is the most important factor in *S*. *pneumoniae* virulence and invasiveness. Around 97 different capsular types (serotypes) have been described and pneumococcal conjugate vaccines (PCVs) developed to date contain the capsular polysaccharides of a limited number of serotypes including those most frequently found to be causing invasive pneumococcal disease (IPD). At present, PCV13 is the conjugate vaccine that covers the most serotypes (1, 3, 4, 5, 6A, 6B, 7F, 9V, 14, 18C, 19F, 19A, and 23F) licensed in Spain for all ages (from 6 weeks of age) to prevent pneumococcal meningitis, sepsis, pneumonia and otitis [13]. The introduction of PCVs led to a reduction in IPD as a consequence of a decrease in the incidence of vaccine serotypes [14,15]. Nevertheless, an undesirable increase in IPD caused by non-vaccine serotypes has also been observed [15–17]. Although the effect of PCVs on OM has not been as widely studied as that on IPD, a similar pattern has been observed, with a decrease in OM caused by vaccine serotypes and an increase in OM caused by non-vaccine serotypes [18–20].

Given the important health and economic problems associated with pneumococcal OM, more epidemiological studies are necessary to improve our knowledge of the effect of pneumococcal conjugate vaccination on OM. Therefore, we conducted a study on cases of pediatric pneumococcal OM in children seen at two referral hospitals in different regions in the north of Spain more than 500 km apart with different vaccination coverage. The main objectives of this study were to determine and explore changes over time in the pneumococcal serotypes causing OM throughout the period 2008–2016 (before and after PCV13) and analyze the impact of PCV13 on OM in these two regions.

## Materials and methods

### Patients and isolates

The study was conducted between 2008 and 2016 at Donostia University Hospital in San Sebastian and at Sant Joan de Deu Hospital in Barcelona. The former hospital handles most of the emergency care and admissions of the pediatric population in the province of Gipuzkoa (around 95,000 children in 2015), while the latter has an estimated pediatric catchment population of 250,000 children <14 years and is a pediatric referral center, which captures around 22% of all pediatric admissions in the region of Catalonia. PCVs were not subsidized by the corresponding public health systems in Gipuzkoa and Barcelona until 2015 and 2016, respectively, but have been available through private providers since the beginning of the study period. The vaccine coverage rate in children younger than 2 years was estimated to be about 70% in Gipuzkoa and 55% in Barcelona in the initial part of the study period, based on the number of vaccine doses sold and published studies [21], rising to coverage rates of >95% once PCVs were included on the child immunization schedule by the corresponding public health systems. In this study, a patient initially vaccinated with PCV7 and then with PCV13 was only considered to have been vaccinated with PCV13.

The study period was divided into three 3-year periods: pre-PCV13 (from 2008 to 2010), early post-PCV13 (from 2011 to 2013) and late post-PCV13 period (from 2014 to 2016).

The study included all *S*. *pneumoniae* isolates cultured from ear discharge swabs of children aged between 0 and 14 years old diagnosed with OM with spontaneous drainage at the emergency room or hospital wards of either of the participating hospitals. Swab samples were collected as part of routine care by pediatricians at the two hospitals and anonymized before starting the study. Samples were sent to the Microbiology Departments where they were processed by Gram-staining and culture. Pneumococci were identified by their colony morphology, the presence of alpha-hemolysis on blood-agar cultures, optochin susceptibility and bile solubility and were prospectively frozen at -80°C for further studies. Other bacteria frequently associated with OM were identified using standard microbiological procedures [11]. Pneumococcal capsular types were determined with multiplex PCR combined with fragment analysis using automated fluorescent capillary electrophoresis [22] and serotypes confirmed with the Quellung reaction.

### Molecular characterization of pneumococcal isolates

Serotype 11A, 19F and 23B pneumococcal isolates were further characterized by multi-locus sequence typing following a protocol published elsewhere (https://pubmlst.org/spneumoniae/).

### Statistical analyses

Differences in the distribution of serotypes were assessed with the chi-square test or Fisher’s exact test when appropriate. Trends over time were analyzed with the chi-square test for trend (GraphPad Instat ver 3.05. La Jolla, CA, USA). A p value of <0.05 was considered statistically significant. Diversity was assessed by calculation of Simpson’s index (using a tool available at http://www.comparingpartitions.info/index.php).

### Ethical considerations

Publication of the results was approved by the Ethics Committee for Clinical Research of the Health Area of Gipuzkoa and by the Ethics Committee of Sant Joan de Déu Hospital. All relevant anonymized data are available within the manuscript and its Supporting Information files.

## Results

Between 2008 and 2016, a bacterial pathogen was identified in 725 (46.1%) of the 1,571 middle-ear exudates collected from children with OM aged ≤14 years old in Gipuzkoa. *S*. *pneumoniae* accounted for 140 (8.9%) of all OM episodes (Table 1), of which samples were available for microbiological studies in 116 cases (83%). During the same period in Barcelona, *S*. *pneumoniae* was identified in 95 (8.8%) of a total of 1,082 middle-ear exudates collected, and samples were available for further studies in 88 (92.6%) cases. A decrease in the rate of *S*. *pneumoniae* OM was observed between the first and second periods (2008–2010 and 2011–2013) in Gipuzkoa (p = 0.02) and Barcelona (p<0.001), while no differences were observed between the second and third periods (2011–2013 and 2014–2016; p = 0.2 in Gipuzkoa and p = 0.5 in Barcelona). There were no differences between the regions in the rates of *S*. *pneumoniae* isolated from the middle-ear exudate cultures of children with OM in any of the three periods (p>0.05 in all cases).

**Table 1:** Temporal distribution of middle-ear exudate cultures performed and pneumococcal positivity in Gipuzkoa and Barcelona, 2008–2016.

	Gipuzkoa			Barcelona			Total		
Years	Total culture	Pneumococcal positive		Total culture	Pneumococcal positive		Total culture	Pneumococcal positive	
	n	n	%	n	n	%	n	n	%
2008–10	189	24	12.7%	175	32	18.3%	364	56	15.4%
2011–13	681	50	7.3%	360	28	7.8%	1041	78	7.5%
2014–16	701	66	9.4%	547	35	6.4%	1248	101	8.1%
Total	1571	140	8.9%	1082	95	8.8%	2653	235	8.9%

Pediatric pneumococcal OM was most frequent in children aged 0 to 2 years (57.8% in Gipuzkoa and 81.8% in Barcelona) (Table 2). First episodes of pneumococcal OM were mainly seen in 1- (26.7%) and 2-year-olds (19.8%) in Gipuzkoa, while in Barcelona, a higher percentage of cases were found in children < 1 year (34.5%) and 1 year old (31%).

**Table 2:** Distribution of pediatric pneumococcal OM episodes in Gipuzkoa and Barcelona by age group. 2008–2016.

	Gipuzkoa				Barcelona				
Age (years)	2008–2010	2011–2013	2014–2016	Total	2008–2010	2011–2013	2014–2016	Total	Total study
0–2	17	22	28	67 (57.8%)	25	21	26	72 (81.8%)	139 (68.1%)
3–4	3	7	10	20 (17.2%)	1	1	6	8 (9.1%)	28 (13.7%)
5–14	4	11	14	29 (25.0%)	2	5	1	8 (9.1%)	37 (18.1%)
Total	24	40	52	116	28	27	33	88	204

Forty (34.5%) children with pneumococcal OM in Gipuzkoa and 51 (58%) in Barcelona had received the PCV (p<0.001).

### Distribution of the serotypes and changes over time

**Fig 1:**
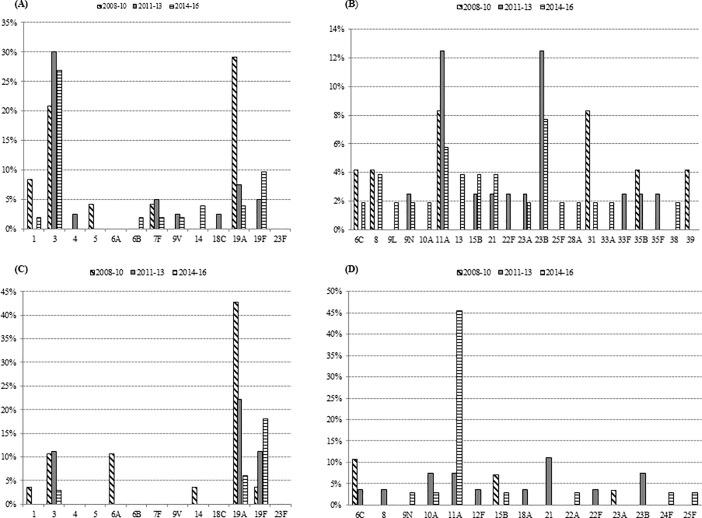
Distribution of serotypes causing OM is two Spanish regions. Percentage of PCV13 serotypes (A) and non-PCV13 serotypes (B) causing OM in each period in Gipuzkoa. Percentage of PCV13 serotypes (C) and non-PCV13 serotypes (D) causing OM in each period in Barcelona.

Taking into account all cases in both regions, the introduction of PCV13 was accompanied by a gradual decrease in the proportion of pneumococcal OM caused by PCV13 serotypes, from 73% in 2008–2010 (pre-PCV13) to 51% in 2011–2013 (early post-PCV13) to 41% in 2014–2016 (late post-PCV13) (p for trend p = 0.001) (Table 3, Fig 1A and Fig 1C). These decreases between the same periods were more evident in Barcelona, from 79% to 44% to 24% (p<0.001) than in Gipuzkoa, where the decrease from 67% to 55% to 52% was less pronounced, not reaching statistical significance (p = 0.48).

**Table 3:** Number of episodes of pediatric pneumococcal OM in Gipuzkoa and Barcelona by serotype, 2008–2016.

Region	Serotype	2008–2010		2011–2013		2014–2016		Total	
		n	%	n	%	n	%	n	%
Gipuzkoa	PCV13	16	(66.7%)	22	(55.0%)	27	(51.9%)	65	(56.0%)
	non-PCV13	8	(33.3%)	18	(45.0%)	25	(48.1%)	51	(44.0%)
Barcelona	PCV13	22	(78.6%)	12	(44.4%)	8	(24.2%)	42	(47.7%)
	non-PCV13	6	(21.4%)	15	(55.6%)	25	(75.8%)	46	(52.3%)
Total	PCV13	38	(73.1%)	34	(50.7%)	35	(41.2%)	107	(52.5%)
	non-PCV13	14	(26.9%)	33	(49.3%)	50	(58.8%)	97	(47.5%)

Three serotypes, 3, 19A and 19F accounted for 87/204 (42.6%) of all pneumococcal OM in both regions (Fig 1A and Fig 1C), but the distribution of serotypes was not the same. Specifically, the percentages of OM attributable to serotypes 19A and 3 were 23.9% and 8% in Barcelona while the rates were nearly the opposite in Gipuzkoa: 10.3% and 26.7%, respectively (p≤0.01 for both serotypes). Serotype 19F accounted for 6% of OM cases in Gipuzkoa and for 10.2% of cases in Barcelona (p = 0.30).

In Gipuzkoa, serotypes 19A and 3 were the most prevalent in the pre-PCV13 period but while the rate of serotype 19A OM declined from 29.2% to 3.8% (p = 0.003) between 2008–2010 and 2014–2016, the rate of serotype 3 remained stable (20.8% and 26.9%, respectively, p = 0.78). In Barcelona, serotype 19A was the most prevalent in the pre-PCV13 period and decreased significantly from 46.4% to 6.1% between 2008–2010 and 2014–2016 (p<0.001), while the prevalence of serotype 3 did not change significantly (10.7% and 3%, respectively, p = 0.325). A surprising increase in OM due to serotype 19F was also observed in both regions, from 1.9% in 2008–2010 to 11.8% in 2014–2016, a difference that nearly reached statistical significance (p = 0.052).

Contrarily, the rates and diversity of non-PCV13 serotypes causing OM increased over the years. Between 2008–2010 and 2014–2016, the number of different serotypes responsible for pneumococcal OM increased from 11 to 24 in Gipuzkoa and from 9 to 13 in Barcelona (Fig 1B and Fig 1D). Simpson’s index showed that serotype diversity increased non-significantly during the study from 0.540 (95% CI 0.363–0.718) to 0.730 (95% CI 0.629–0.830) in Gipuzkoa and from 0.570 (95% CI 0.369–0.771) to 0.657 (95% CI 0.540–0.773) in Barcelona.

Among non-PCV13 serotypes, a large increase was observed in the rate of one specific serotype in each region. In Barcelona, there was a marked increase in serotype 11A OM, from 0% in 2008–2010 to 45.5% in 2014–2016 (p<0.005), while in Gipuzkoa, OM caused by serotype 23B increased after 2010, this having been the most frequent non-PCV13 serotype since 2011 (16% of all non-PCV13 serotypes). Interestingly, in Barcelona, most 11A OM episodes occurred in vaccinated children, whereas most cases of 23B infection occurred in unvaccinated children (Table 4).

**Table 4:** Distribution (number) by serotypes and vaccination status of the episodes of pneumococcal OM in Gipuzkoa and Barcelona. 2008–2016.

	Gipuzkoa							Barcelona						
Serotype	Vaccinated			Non-vaccinated				Vaccinated			Non-vaccinated			
PCV13	2008–2010	2011–2013	2014–2016	2008–2010	2011–2013	2014–2016	Total	2008–2010	2011–2013	2014–2016	2008–2010	2011–2013	2014–2016	Total
1				2		1	3				1			1
3		2	3	5	10	11	31	2	1		1	2	1	7
4		1					1							0
5				1			1							0
6A							0	1			2			3
6B			1				1							0
7F		2	1	1			4							0
9V		1	1				2							0
14			2				2				1			1
18C		1					1							0
19A	2	2	2	5	1		12	7	3	1	6	3	1	21
19F		2	2			2	6		1	1	1	2	4	9
23F							0							0
Total PCV13	2	11	12	14	11	14	64	10	5	2	12	7	6	42
Non-PCV13														
11A		1	2	2	4	1	10		2	12			3	17
23B			1		5	4	10		1			1		2
others	1	2	8	5	6	10	32	4	6	9	2	5	1	27
Total non-PCV13	1	3	11	7	15	15	52	4	9	21	2	6	4	46

### Clonal characterization of serotypes 11A, 19F and 23B

In Gipuzkoa, the number of serotype 19F OM episodes gradually rose from 0 in 2008–2010 to 2 cases in 2011–2013 to 5 cases in 2014–2016. The 2 cases in 2011–2013 and 4 of the 5 cases in 2014–2016 were ST179 isolates (Table 5), while the other case in 2014–2016 was ST5216. The same trend was observed in Barcelona, with only 1 serotype 19F OM episode in 2008–2010 (ST1545), 3 in 2011–2013 (two ST179, one ST15) and 5 (four ST179, one ST51) in 2014–2016. Of the 16 serotype 19F cases, 6 occurred in children fully vaccinated with PCV13.

**Table 5:** Clonal distribution of OM cases caused by *S*. *pneumoniae* serotypes 19F, 11A and 23B in Gipuzkoa and Barcelona.

Period	Gipuzkoa		Barcelona	
	serotype 19F	ST179	serotype 19F	ST179
2008–2010	0	0	1	0
2011–2013	2	2	3	2
2014–2016	5	4	5	4
	serotype 11A	ST838/ST6521	serotype 11A	ST838/6521
2008–2010	2	0/1	0	0/0
2011–2013	5	0/1	2	0/1
2014–2016	3	0/2	15	5/9
	serotype 23B	ST2372	serotype 23B	ST2372
2008–2010	0	0	0	0
2011–2013	5	5	2	2
2014–2016	4	4	0	0

In Barcelona, a dramatic increase from 0 to 15 cases of serotype 11A OM was also observed between 2008–2010 and 2014–2016. Of the isolates from the 2 cases in 2011–2013, 1 was identified as ST6521 and the other as ST62, while the 15 episodes recorded in 2014–2016 were classified as ST6521 (n = 9), ST838 (n = 5) and ST3687 (n = 1). In Gipuzkoa, the 2 cases of serotype 11A OM in 2008–2010 were identified as ST6521 and ST62, while the 8 serotype 11A OM cases since 2010 have been identified as ST62 (n = 5) and ST6521 (n = 3).

Serotype 23B was not detected in OM in Gipuzkoa until February 2011, but became the most frequent among non-PCV13 serotypes in 2011–2013 and 2014–2016. All serotype 23B isolates from both regions were identified as ST2372.

### Vaccination status and serotypes

Taking into account all cases from both regions, the proportion of PCV13 serotypes decreased after PCV13 introduction: from 12/17 (70.6%) episodes in 2008–2010 to 30/74 (40.5%) in 2011–2016 (p = 0.03) in vaccinated children and from 26/35 (74.3%) to 39/78 (50%) in unvaccinated children for the same periods (p = 0.02).

Since 2011, in vaccinated children, the proportion of PCV13 serotypes causing OM has been higher in Gipuzkoa (23/37, 62.2%) than in Barcelona (7/37, 18.9%, p<0.001) (Table 4). The high proportion of PCV13 serotypes in vaccinated children in Gipuzkoa is attributable to several PCV13 serotypes, without the emergence of a particular serotype. In Gipuzkoa, since 2011, the proportion of serotype 3 OM infections was lower in vaccinated (5/37, 13.5%) than in unvaccinated (21/55, 38.2%) children (p = 0.01).

In unvaccinated children since 2011, however, no difference in the distribution of PCV13 serotypes was observed between Gipuzkoa and Barcelona: 26/55 (47.3%) and 13/23 (56.5%), respectively (p = 0.62). Most cases were due to serotype 3 (21/26) in Gipuzkoa and to serotypes 19F and 19A (10/13 episodes) in Barcelona.

### Reinfections

Both in Gipuzkoa and in Barcelona, 3 children suffered reinfections representing 2.7% and 3.5% of all OM cases, respectively, 3.3% and 5% considering only the post-PCV13 period (2011–2016). All 6 reinfections were attributable to an isolate with a different serotype to that causing the first episode, except in one patient who suffered two episodes caused by the same serotype 11A but different genotypes ST838 and ST6521 belonging to the same clonal complex (Table 6).

**Table 6:** Serotypes and vaccination status of reinfection episodes.

Region	Patient ^a^	First episode	Reinfection episode	Year of episode First/reinfection	Time (days) between episodes
Gipuzkoa	1	23B	15B	2014/2015	308
	2^a^	3	11A	2012/2016	1177
	3	19A	8	2012/2015	1000
Barcelona	1^a^	11A	11A	2014/2014	196
	2^a^	11A	25F	2014/2015	76
	3	23B	21	2012/2012	279

^*a*^ Patients vaccinated with PCV13

### Mixed Infections

Of the 204 episodes of OM studied, 54 (26.5%) corresponded to mixed infections (Table 7). Of them, 37 (31.9%) occurred in Gipuzkoa and 17 (19.3%) in Barcelona, with *H*. *influenzae* (44/54, 81.5%) being the most frequent microorganism isolated together with *S*. *pneumoniae*. Serotypes 3 (9/54, 16.7%), 11A (9/54, 16.7%) and 23B (6/54, 11.1%) were the most prevalent in mixed infections. In fact, serotype 23B was more frequently found in mixed infections (6 cases) than alone (5 cases).

**Table 7:** Number (percentage) of pneumococcal single and mixed OM infections in Gipuzkoa and Barcelona.

	Gipuzkoa	Barcelona	Total
*S*. *pneumoniae* alone	79 (68.1)	71 (80.7)	150 (73.5)
*S*. *pneumoniae* and *H*. *influenzae*	21 (18.1)	16 (18.2)	37 (18.1)
*S*. *pneumoniae* and *H*. *influenzae* and other bacteria	6 (5.2)	1 (1.1%)	7 (3.4%)
*S*. *pneumoniae* and other bacteria	10 (8.6)	0 (0)	10 (4.9)
Total	116 (100)	88 (100)	204 (100)

## Discussion

At present, PCV13 is the pneumococcal conjugate vaccine in use that covers the largest number of serotypes causing invasive diseases [23]. IPD constitutes a group of severe infectious diseases but there are other milder pneumococcal infections, like OM, that are also a major health and economic burden. Several research studies have shown that PCV13 protects from pneumococcal OM and from complications such as reinfections [9]. Nevertheless, the fact that not all pneumococcal serotypes can be included in a single conjugate vaccine makes it necessary to conduct studies determining the distribution of serotypes causing human infections.

In this study, the decision to send an ear exudate for culture when the presence of infection was suspected was made by the treating pediatricians. In Gipuzkoa, the demand for OM diagnosis increased 60–70% between 2008–2010 and 2011–2013 without a plausible explanation, as the rates of total bacterial pathogens identified by culture remained steady and no outbreaks of any particular pathogen were observed (data not shown). Consequently, the difference in the absolute number of *S*. *pneumoniae* isolated between periods in Gipuzkoa cannot be attributable to an increase in pneumococcal OM infections but rather to an increase in the demand for microbiological analysis. In fact, the rate of *S*. *pneumoniae* positive cultures decreased after the introduction of PCV13.

In Barcelona, the percentage of children with OM that had been vaccinated was 58%, similar to the figure of 55% reported for the general population [24]. Contrarily, in Gipuzkoa, the percentage of vaccinated children with OM was 34.5%, nearly half of the 70% coverage estimated for the population overall. Another difference between the two regions was the higher prevalence of OM caused by serotype 3 in Gipuzkoa and by serotype 19A in Barcelona. It is plausible that other factors, such as serotype prevalence or specific clones circulating at the time of introducing PCV13, could have influenced the effect of the vaccine in each region.

In Gipuzkoa, after PCV13 introduction, the decrease in OM caused by PCV13 serotypes did not reach statistical significance because of the persistence of serotype 3 and despite the dramatic decrease in serotype 19A. A similar trend for serotypes 3 (persistence) and 19A (decrease) causing OM has been previously described by other authors [25,26]. The persistence of serotype 3 infections in Gipuzkoa was especially evident in unvaccinated children because, unlike in other studies [27,28], a lower rate of serotype 3 OM was observed in vaccinated children.

In Barcelona, however, a very significant decrease in PCV13 serotypes was observed as seen in preceding surveys [26], a decrease that paralleled the decrease in serotype 19A. The near disappearance of serotype 19A among unvaccinated children from both regions could be due to the herd protection effect of PCV13, an effect not observed for serotype 3 in Gipuzkoa.

The association of serotype 19F with OM and the weak protection of PCVs against OM caused by this serotype have been described previously [11,29,30]. After PCV7 introduction in Gipuzkoa, a large drop was observed in the prevalence serotype 19F [12]. The slight increase in serotype 19F incidence in Gipuzkoa and Barcelona was due to ST179 isolates that proved to be a successful clone causing OM even in PCV13-vaccinated children.

After PCV13 introduction, two similar epidemiological changes in non-PCV13 serotypes were observed in both regions: an increase in the rate and diversity of non-PCV13 serotypes or serotype replacement, a well described phenomenon in IPD and OM [20,24] and a sharp increase in a different single serotype in each region. In Barcelona, in the post-vaccine period there was a dramatic increase in OM caused by serotype 11A, ST838 and ST6521 (all penicillin-resistant, data not shown). ST838 and ST6521 share six of the seven alleles differing in two nucleotides at the *aroE* allele and are SLV and DLV (double-locus variant) of ST156, respectively. Since 2010, a marked increase in a penicillin-resistant serotype 11A ST838/ST6521 clone has been observed among invasive Spanish isolates [31]. First detected in Spain in 2005, ST838/ST6521 is a SLV/DLV capsular variant of the successful Spain^9V^-156 clone, a worldwide disseminated penicillin-resistant clone before PCV7 introduction [31]. This finding underscores the need for close monitoring of not only the serotypes, but also the genotypes of all types of pneumococcal infections over time.

In Gipuzkoa, an increase in serotype 23B ST2372 has been observed since 2011, coinciding in time with the introduction of PCV13, and more frequently found in unvaccinated children with mixed OM. Serotype 23B ST2372, also observed in two isolates from Barcelona, was first found among 23B invasive isolates in the UK [32]. Surprisingly, in the UK, ST2372 was described as a genotypic subtype of serotype 23B, with the same capsular polysaccharide structure and immune reactivity as serotype 23B but with <70% similarity to the reference 23B capsular cassette sequence. In Gipuzkoa, as well as in UK, ST2372 prevalence increased after 2010. The concurrence in time of the appearance of ST2372 in Gipuzkoa, Barcelona and UK indicates a local appearance of this clone derived from isolates already present in each region.

Considering the entire post-PCV13 period, a decrease in the percentage of pneumococcal reinfections in patients with a first pneumococcal OM, from 12.1% (1999–2010) to 3.3% (2011–2016) was observed in Gipuzkoa [2]. It has been reported that prevention of first episodes of OM caused by vaccine serotypes reduces recurrences and chronic cases [9]. Besides, the composition of microbiota differs greatly between PCV7-vaccinated and non-vaccinated children [33]. Hence, changes in nasopharyngeal microbiota generated by pneumococcal vaccination might reduce the risk of later complex pneumococcal OM as observed in our study.

Overall, in nearly one third of pneumococcal OM a second pathogen was isolated together with *S*. *pneumoniae*, a similar rate to previous studies [12] with *H*. *influenza*e again being the most frequent microorganism found in mixed infections.

The results herein reported corroborate earlier observations such as the reduction in OM caused by vaccine serotypes after PCV introduction [9], and the weak protection against serotypes 3 and 19F OM [6,11, 26,29]. Moreover, this study shows the spread of serotype 11A causing OM in Spain as has already been described in invasive disease [31] and the spread and association of the international serotype 23B ST2372 clone with mixed OM in Gipuzkoa. Several differences in the epidemiology of OM between the two regions were also observed, in particular, the higher rate of sample collection by pediatricians in Gipuzkoa; the lower rate of vaccinated children with pneumococcal OM in Gipuzkoa; the different distribution of PCV13 serotypes, with lower serotype 3 but higher serotype 19A OM rates in Barcelona; and the differently successful spread of serotype 11A in Barcelona and 23B in Gipuzkoa. Until a pneumococcal vaccine capable of protecting against all pneumococcal serotypes becomes available, all these observations reflect the importance of continuous epidemiological studies to analyze the effect of conjugate vaccines on non-invasive pneumococcal infections.

## Supporting information

S1 File Original de-identified data of otitis cases. (XLSX)
